# Insights from the molecular docking analysis of phytohormone reveal brassinolide interaction with HSC70 from Pennisetum glaucum

**DOI:** 10.6026/97320630015131

**Published:** 2019-02-28

**Authors:** Gugulothu Baloji, Shobharani Pasham, Vinodha Mahankali, Mallikarjuna Garladinne, Srinivas Ankanagari

**Affiliations:** 1 Department of Genetics and Biotechnology, Osmania University,Hyderabad (T.S) - 500 007,India; 2 Plant Molecular Biology Laboratory,Agri Biotech Foundation,Rajendra Nagar,Hyderabad (T.S) 500 030,India;

**Keywords:** heat stress, HSP70/HSC70, phytohormones, heat tolerance

## Abstract

The prevailing abiotic stresses, especially heat stress is of great significance on the growth of plants, yield and distribution. In the conditions of heat stress, plants modulate protein processes leading to development of heat tolerance. Of such proteins, the molecular chaperone functions of HSP70/HSC70 proteins are important where their enhanced expression positively correlates with the acquisition of heat tolerance. The key players in the regulation of such tailored protein responses of plants to heat stress are the phytohormones. In the present study, phytohormone mediated interaction of Pennisetum glaucum HSC70 (PgHSC70) protein was performed through docking studies involving sequence analysis, 3D modeling and model evaluation. In silico analysis has shown better interaction and good binding energy of PgHSC70 with the phytohormone brassinolide. Furthermore, the predicted structural information can be helpful for future studies on role of interaction between HSC70 and brassinolide in heat tolerance.

## Background

As plants being sessile organisms, they are endlessly exposed to diverse climatic conditions like drought, salt, heat, flooding, and oxidative stress. The main abiotic stress, heat, essentially leads to protein dysfunction, which ultimately impacts the growth of plants, harvest, and distribution [1-5]. Heat stress lead to serious effects on protein metabolism, together with inhibition of protein accumulation, depravity of proteins, and initiation of certain protein synthesis, depending on the amount and duration of heat stress [6,7]. Conservative heat responses include down-regulation of proteins functioning in cytoskeleton structure, lipid biogenesis, amino acid biosynthesis, sulfate assimilation, antioxidant response, and nuclear transport [8,9]. Moreover, the synthesis of mRNAs and most of the normal proteins is constrained under heat stress conditions. The transcription and translation of the heat shock proteins (HSPs) may either be improved or induced when plants are exposed to increased temperatures [10, 11, 12]. Heat-shock proteins (HSPs) function as molecular chaperones in order to expedite the refolding of impaired proteins, hinder irreversible protein aggregation and keep up cellular homeostasis under both unfavorable and optimum developmental conditions. HSPs are found to be up-regulated on exposure to higher temperatures along with other various environmental and physiological stress factors like cold, anoxia, metal drought [9], and salinity [10] and guard protein structure and function. They aid in varied functions but are specifically associated with acquired heat tolerance. Refined upregulation and over expression of HSP70 positively equates with the acquisition of heat tolerance [13-16].

Based on the approximate molecular weights, five extensive families of HSPs are recognized: Hsp100, Hsp90, Hsp70, Hsp60 and the small HSP (sHSP) families. Among these groups, HSP70 family chaperons with a molecular weight of 70kDa are highly conserved family of heat inducible (HSP70s) and constitutively expressed (HSC70s) proteins in different organisms and in different cellular structures in the same organism [17, 18, 19]. In plants, the HSP70/HSC70 family consists of members located in the mitochondrion, plastid, endoplasmic reticulum and cytosol [17]. Distinct expression of HSC70s/HSP70s has been reported in various tissues during different developmental stages. All HSP70 proteins in higher eukaryotes along with plants share analogous structure, consisting of an N-terminal nucleotide-binding domain (NBD) that exhibit modest ATPase activity on its own, and a Cterminal peptide substrate-binding domain (SBD). The functions of the two main domains of the HSP70 proteins are allosterically controlled. The SBD and NBD are linked by a highly conserved inter-domain linker (also known as loop LL1), which is critically involved in inflecting the allosteric regulation of HSP70 proteins. The extreme C-terminal domain of HSP70 is assumed to be broadly unstructured and is the docking site for few co-chaperones [19-23, 24-26].

One of the substantial and broadly conferring endogenous messenger molecules to abiotic stress tolerance are phytohormones that play a crucial role in plant growth and development [27, 28, 29]. Numerous reports documented evidence about the phytohormones active involvement in physiological protection against heat stress. The adaptation of plants to abiotic stresses illustrates the potentiality of phytohormones in the refinement of stress responses in plants [30, 31, 32]. Taking into account the phytohormone mediated heat stress tolerance; present study investigated the involvement of phytohormone interaction with the up-regulated HSC70 protein of Pennisetum glaucum. In this study, sequence analysis, 3D structure modelling and validation of the target protein HSC70 were carried out. Furthermore, molecular docking was applied to study binding conformations and structural specificity of the selected phytohormones. The phytohormone mediated interaction of PgHSC70 protein through in silico analysis showed good binding energy with brassinolide

## Methodology

### Sequence retrieval and analysis:

The amino acid sequence of the HSC70 from Pennisetum glaucum was retrieved from the SwissProt database using the accession No C7E6Z5. The primary structure was predicted using ProtParam tool. The physicochemical properties were computed that included the molecular weight, theoretical isoelectric point (pI), amino acid composition, atomic composition, total number of positive and negative residues, extinction coefficient, estimated half-life, instability index, aliphatic index, and grand average of hydropathicity (GRAVY). The secondary structure of this protein was predicted using SOPMA (Self Optimized Prediction Method with Alignment) program that uses multiple alignments. It calculated and analyzed the secondary structural features alpha helixes, extended strand, random coil and beta turns of the protein sequence.

### 3D Structure modelling:

The 3D structure of the protein HSC70 from Pennisetum glaucum was determined using the homology modelling methods of Discovery Studio (DS). Homology modelling was for the alignment of the target sequence and the sequences of known structures of one or more proteins as templates, which resembled the structure of the query sequence. The sequence alignment and template structure are then used to generate a structural model of the target. The retrieved FASTA sequence as query, using BLAST against PDB, the homologous sequences with known related protein 3D structures was used as templates search. From the obtained BLAST results, the sequence with maximum identity score and lowest evalue was retrieved. The query and the template sequences are aligned using align sequences program of DS. Based on this sequence alignment, using crystal structural coordinates of templates; three 3D models of target protein were generated with the homology model-building program of DS Modeler. The best model of target was selected on the basis of the results of the internal scoring functions analysis, PDF (Probability Density Functions) total energy, PDF physical energy and DOPE scores of DS. The best homology model with least energy function was taken for further refinement using DS energy minimization methods. At first, all the hydrogen atoms were allowed while performing the calculations. Energy minimization was carried out under CHARMM force fields using 1000 steps of the steepest descent algorithm followed by 1000 steps of the conjugate gradient algorithm, with minimization criteria set at 0.001 root mean square gradient, respectively to obtain a stable and low energy conformation. After the optimization procedure, the final 3D model was chosen for further validation.

### Model Evaluation:

The final 3D model of PgHSC70 was chosen for validation using RAPPER, ProSA and Root Mean Square Deviation (RMSD). The modelled structure validated by RAPPER was verified with Ramachandran plot through analysis of the possible conformations of dihedral angles Ψ and Φ of amino acid residues in protein structure for the assessment of the stereo chemical quality. ProSA was used to analyze the energy criteria and the Z-score values for the comparison of reliability. The model Z score was compared to the Z scores determined experimentally of similar structures. Additionally, the Root Mean Square Deviation value, which indicates degree of similarity of the 3D structures, was done both for the query and template structures. The structures were superimposed for RMSD calculation using SPDBV program. Lower RMSD value represented more similarity in the structures. Finally, the best quality model of PgHSC70 was subjected to further calculations and molecular modelling studies.

### Identification of Active Site:

The possible binding site of the modelled PgHSC70 were predicted according to the receptor cavity method (Eraser algorithm) using the DS Analyze Binding Site tool. The receptor molecule was first defined by using 'define receptor molecule module' of DS. The protein active site-Search was done using 'find sites from the receptor cavities' which identified the protein active sites by locating cavity in the modelled structure. When the search was completed, the largest site, Site 1 was automatically displayed on the structure. The predicted site 1 was chosen as the most favourable binding site for docking with the ligands.

### Generation of ligand dataset:

**Figure 1:**
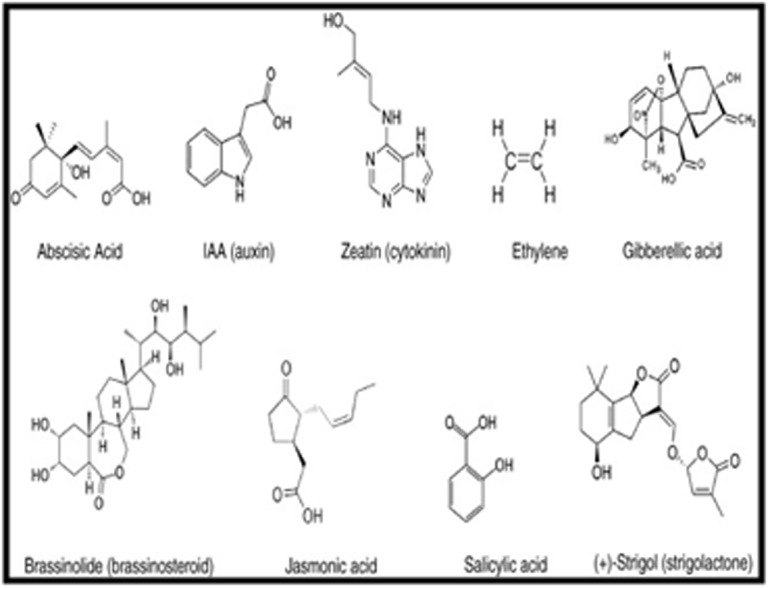
Chemical structures of phytohormones used for ligand preparation

All the chemical structures of the selected phytohormones shown in (Figure 1) were drawn using ACD/ChemSketch (12.0) and later imported in DS for ligand preparation. Ligands were prepared using Prepare Ligands protocol which added hydrogen, generated all possible stereoisomer's, ionization and tautomeric states, converted 2D to 3D structures using catalyst algorithm and minimized the energy of ligands through CHARMM force field using smart minimizer algorithm with the default settings that was continued by steepest descent and conjugate gradient algorithm until the compounds reached with a convergence gradient of 0.001 kcal/mol.

### Molecular docking:

Molecular docking studies were carried out to predict the interaction energy and best orientation between the target protein and ligand molecules. The docking program LibDock provided by DS was used for high-throughput site-featured algorithm to dock ligands into a receptor binding site. LibDock used protein polar and apolar interaction site features, referred to as hot spots, where the ligand aligned to form favourable interaction. All other docking and consequent scoring parameters used were kept at their default settings. The docking calculation generated few minimized poses for each ligand. The selection of the best pose was done on the interaction energy between the ligand and the protein and interactions with the important residues of the protein. Each pose was evaluated according to the LibDock score which was calculated using a simple pair-wise method and the ligands with top LibDock scores were selected for calculation of binding energy between the receptor and ligand. The complex pose with the best binding energy was used for further binding mode analysis. Further, the hydrogen bond formed between the protein-ligand complex and the receptor docking conformation was calculated with the 'Analyze Ligand Poses' process analysis.

## Results and Discussion:

### Sequence retrieval and analysis:

The sequence of PgHSC70 was retrieved in FASTA format from SWISS PROT database with Accession No: G0254653 and entry name: C7E6Z5_PENAM. The primary structure was predicted using ProtParam tool and the physicochemical parameters computed are presented in (Table 1). Results showed that the protein has 649 amino acid residues with an estimated molecular weight of 71105.48 daltons. The maximum number of amino acids present in the sequence was found to be Ala (8.9%) and least was that of Trp (0.5%). The atomic composition was C2777H4999N861O995S24. The total number of negatively charged residues (Asp+Glu) are 100 and the total number of positively charged residues (Arg+Lys) are 82. The isoelectric point pI was 5.10 revealing the basic nature of this protein. The instability index is 34.52 which classify the protein as stable and the aliphatic index is 81.79 which indicate that this protein is thermostable. At 280 nm, the protein's extinction coefficient was evaluated and the value was 37735. The estimated half-life is 30 hours (mammalian reticulocytes, in vitro), greater than 20 hours (yeast, in vivo) and greater than 10 hours (Escherichia coli, in vivo). Negative GRAVY value indicates the hydrophilicity of the protein. The calculated GRAVY of -0.427 indicates that this protein is hydrophilic and soluble in nature.

**Figure 2:**
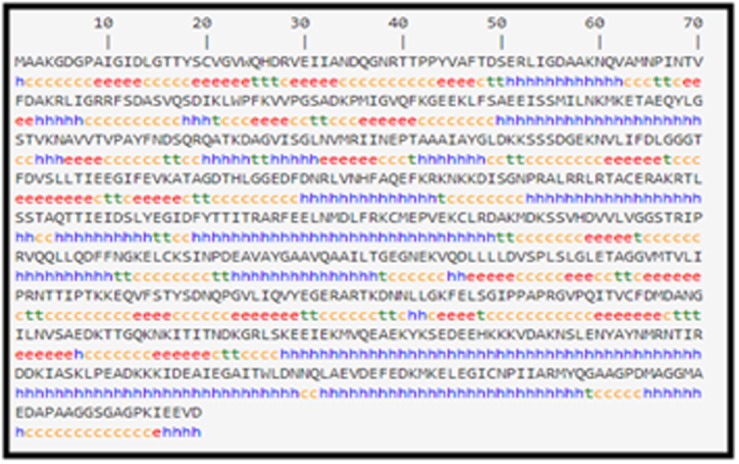
Secondary structure of PgHSC70 predicted by SOPMA

Secondary structure predicted by SOPMA program is shown in (Figure 2) and the results of the analysis are shown in (Table 2). Alpha helix was predominant (42.68%), followed by random coil (31.90%) and extended strand (18.03%). Also, beta turn was found as 7.40%. The high percentage of random coils indicates the flexibility of the protein which is responsible for more interactions.

### 3D Structure modelling:

**Figure 3:**
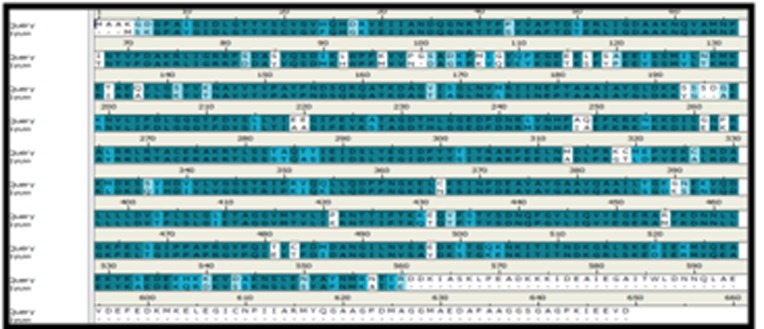
Alignment between the query sequence PgHSC70 and the template 1YUW. Thick blue color represents the conserved regions and light blue color represents variable regions of template and query.

**Figure 4:**
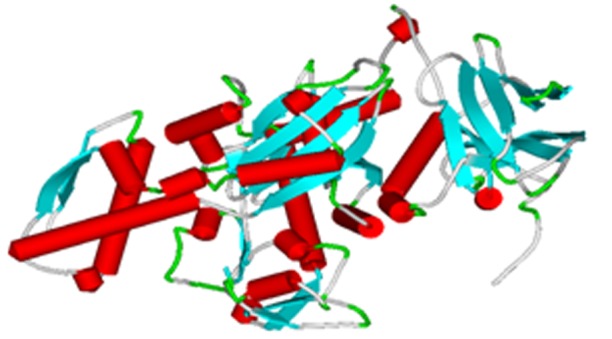
Modelled structure of PgHSC70 consisting of 22 helices,38 strands and 57 turns

 Homology modelling of PgHSC70 was predicted based on the BLAST search against the PDB database for homologous template. From the BLAST results, the selected template was the crystal structure of a bovine HSC70 (PDB ID: 1YUW) (amino acids: 1-554, resolution 2.6 Å), having 81% identity and 83% similarity with the target protein. A 3D homology model of PgHSC70 was built on the basis of sequence alignment of the selected template (1YUW) and target protein PgHSC70 as shown in (Figure 3). The protocol 'Align multiple sequences' of DS performed sequence alignment and the initial models of the PgHSC70 was built using protein modelling protocol called 'build homology model' which used modeller to build homology models. The modeller options were kept default while running and ended up with a loop refinement by default modelling process. Of the three generated models, the model with least energy function was chosen. It has the lowest value in PDF total energy (6141.58), PDF physical energy (-556.69) and DOPE (Discrete Optimized Protein Energy) score (-40198.65), that indicated it as the best model. The energy refinement method has given the best conformation to the model. For optimization, the CHARMM force field and steepest descent algorithm was applied with 0.001 minimizing RMS gradient and 1000 minimizing steps. Following the steps of minimization, the protein was minimized using conjugate gradient algorithm preceded by smart minimizer algorithm until the convergence gradient of 0.001 kcal mol-1 was satisfied. The finally refined protein generated is shown in (Figure 4).

### Model Validation:

The refined 3D structure of PgHSC70 was validated through the RAPPER for Ramchandran plot analysis, ProSA for energy criteria analysis and Root mean square deviation (RMSD) of template and PgHSC70 model using SPDBV. The stereo-chemical quality and accuracy of the final refined modelled PgHSC70 protein was evaluated by Ramachandran plot calculations. The model showed that 94.6% residues are in favoured region, 4.4% residues in the allowed region and 1.1% residues in the outlier region. Of the total sequence length, 521 amino acid residues are in favourable regions and 24 residues in allowed regions and only five residues in outliner region indicating the energetically and sterically stable conformations of residues characterized by values of torsion angles Ψ and Φ.

**Figure 5:**
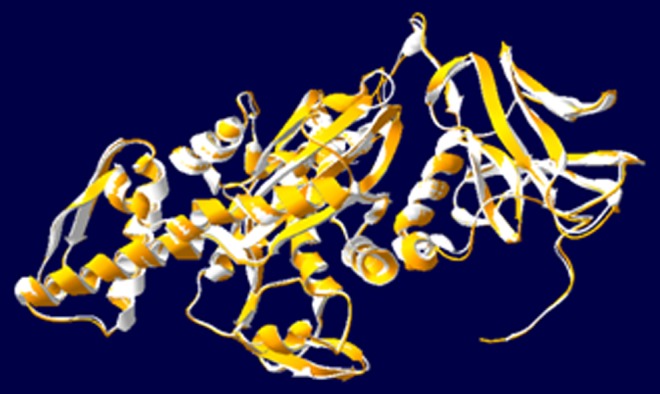
RMSD (0.62 Å) between query PgHSC70 (white) and template 1YUW (orange).

ProSA was used to analyze the energy criteria and the Z-score values for the comparison of reliability. ProSA reveals that the overall quality of the predicted PgHSC70 model by comparing the potential of mean forces derived from a large set of known NMR and X-ray deciphered structures of similar sizes and group. The model quality assessment of Z score is -10.38 kcal/mol for PgHSC70 model suggesting the model being within the permissible range of native conformational structures. Finally, the RMSD value indicates the degree of structural similarity of the template 1YUW and PgHSC70, calculated by SPDBV program. In this, both the query and template structures were superimposed (Figure 5) for RMSD calculation, which is 0.62 Å.

### Molecular docking:

**Figure 6:**
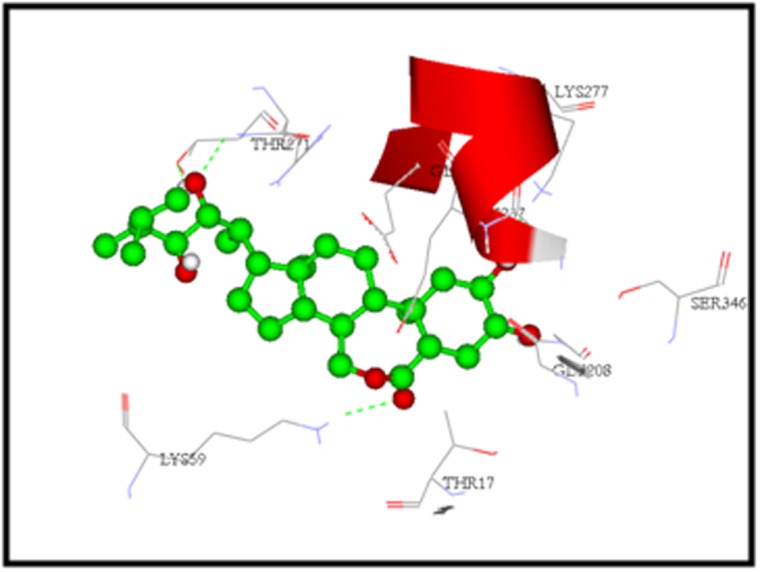
Hydrogen bond interactions of Brassinolide with PgHSC70. Green dotted lines represent hydrogen bonds.

In an attempt to further corroborate the phytohormones, aimed at better modulation of PgHSC70 activity, docking studies were investigated using the LibDock docking program. From the docking studies, both the binding affinities and hydrogen bond interactions were generated which were employed as the criterion for ranking the docked complexes of the protein and the ligand. Each docked conformation was assigned a LibDock score according to its binding mode onto the binding site. Of all the conformations generated for each compound, the compound with the highest LibDock score was taken for interaction analysis of the hydrogen bonding. LibDock scores of all the compounds along with their hydrogen bond interactions and bond lengths are depicted in the (Table 3). Finally, the 'Analyze Ligand Poses' sub protocol was performed to count H- bonds and close contacts (van der Waals clashes) between the poses and PgHSC70. The molecular docking simulation study revealed that the binding mode of brassinolide shows high LibDock score of 115.231 K.cal/mol and forms five hydrogen bonds with the amino acids LYS59, THR271, LYS277. (Figure 6), show the amino acid residues involved in hydrogen bond interactions with PgHSC70 and brassinolide. The hydrogen bonds are formed between the hydrogen atom of LYS59 interacting with the oxygen atom of the Brassinolide (LYS59:HZ1- Brassinolide:O12) with a distance of 2.180000 Å, between nitrogen atom of THR271 interacting with oxygen atom of the Brassinolide (THR271:HN- Brassinolide:O33) with a distance of 2.348000 Å, between hydrogen atom of THR271 interacting with oxygen atom of the Brassinolide (THR271:HG1Brassinolide:O33) with a distance of 2.381000 Å, between the hydrogen atom of LYS277 interacting with the oxygen atom of the Brassinolide (LYS277:HZ1- Brassinolide:O29) with a distance of 2.284000 Å and between the hydrogen atom of Brassinolide interacting with the oxygen atom of the THR271 with a distance of 2.196000 Å. It shows some close contacts with the amino acid residue GLY236.

## Conclusion

Phytohormones are the key players in the regulation of molecular chaperons/HSP protein (HSP/HSC70) responses under heat stress. The current study focused on the interaction of phytohormones with the protein PgHSC70. The molecular docking simulation study revealed that the binding mode of brassinolide has high LibDock score of 115.231 K.cal/mol and forms five hydrogen bonds with the amino acids LYS59, THR271, LYS277 showing better binding energy and good interactions compared to other hormones. Findings of this study on the predicted structural information suggest the role of interaction between HSC 70 and brassinolide. Further in vivo studies are needed to confirm the efficacy of this interaction in heat tolerance.

## Conflict of Interest

none

## Figures and Tables

**Table 1:** predicted physicochemical properties of the PgHSC70

Parameter	Value
Amino Acid Length	649
Molecular Weight (M.wt.)	71105.48
pI	5.1
Total number of negatively charged residues (Asp + Glu)	100
Total number of positively charged residues (Arg + Lys)	82
Instability index (II)	34.52
Aliphatic index (AI)	81.79
	Mammalian reticulocytes	30 hours
Half-life	Yeast	>20 hours
	E. coli	>10 hours
GRAVY	-0.427

**Table 2:** Secondary structure analysis of PgHSC70 by SOPMA

Parameters	Number of amino acids	Amino acids (%)
Alpha helix (Hh)	277	42.68
310 helix (Gg)	0	0
Pi helix (Ii)	0	0
Beta bridge (Bb)	0	0
Extended strand (Ee)	117	18.03
Beta turn (Tt)	48	7.4
Bend region (Ss)	0	0
Random coil (Cc)	207	31.9
Ambiguous states	0	0
Other states	0	0

**Table 3:** Calculated docking scores and interacting amino acids for phytohormones in the active site of modelled PgHSC70.

Name	LibDockScore(K. cal/mol)	Interacting amino acids	H-bond distance(in Å)	Binding energy(K. cal/mol)
:ARG75:HE - Abscisic acid:O18	1.745000	0.00118
:ARG75:HH21 - Abscisic acid:O16	2.459000	
Abscisic acid	80.887	:ARG75:HH21 - Abscisic acid:O18	2.252000 1.956000
Abscisic acid:H36 - :ASP72:OD2	1.943000 1.512000
Abscisic acid:H27 - :ASP72:OD2 Abscisic acid:H36 - :ARG75:HE
LYS59:HZ1- Brassinolide:O12
THR271:HN- Brassinolide:O33
THR271:HG1Brassinolide:O33: LYS277:HZ1 - Brassinolide:O29
Brassinolide:H79 - :THR271:OG1	2.18000
Brassinolide	115.231	Brassinolide:H45 - :LYS59:CE	2.348000 2.381000 2.284000 2.196000 2.101000 2.071000
Brassinolide:H45 - :LYS59:NZ	1.458000	0.00119
Brassinolide:H45 - :LYS59:HZ1	2.123000 1.584000 1.651000
Brassinolide:H71 - :GLY236:CA
Brassinolide:H78 - :THR271:HG1
Brassinolide:H79 - :THR271:HG1
Ethylene	-	0.00118
:ARG75:HE-Gibberellic acid:O13	1.8990002.317000 1.944000 2.026000
ARG75:HH21-Gibberellic acid:O12	1.905000	0.00119
Gibberellic acid	98.633	Gibberellic acid:H43- :ASP238:OD1 Gibberellic acid:H43- :ASP238:OD2	1.916000	
Gibberellicacid:H35- :GLU237:OE1		
Gibberellic acid:H45- :ASP238:OD1		
:TYR18:HH - IAA:O13	1.600000 2.342000 1.943000	0.00119
:SER346:HN - IAA:O12	1.872000 2.172000
IAA72.354:SER346:HG - IAA:O12
:GLU237:CG - IAA:H17
:SER346:OG - IAA:O12
:TYR18:HH - Jasmonic acid:O14
Jasmonic acid84.199:LYS277:HZ1 - Jasmonic acid:O6 :SER346:HN - Jasmonic acid:O15	2.063000 2.353000	0.00119
Jasmonic acid:H16 - :ARG278:CG	2.429000 2.147000	
:LYS277:HZ1 - Salicylic acid:O8
:LYS277:HZ1 - Salicylic acid:O9
Salicylic acid:H15 - :ASP240:OD1 Salicylic acid:H15 -	2.025000 2.186000 2.459000 2.291000 2.108000	0.00119
:ASP240:OD2
Salicylic acid:C7 - :LYS277:HZ1
:LYS59:HZ1 - Strigolactone:O4		0.00119
Strigolactone96.732Strigolactone:H41 - :ASP240:OD2	2.075000 1.814000 1.519000	
Strigolactone:H47 - :LYS59:HZ1
Zeatin82.88A:LYS59:HZ1-Zeatin:N4	2.028000	-0.26316
A :LYS59:HZ3 - Zeatin:N4	2.040000
A :LYS59:HZ3 - Zeatin:N9	2.494000
A:LYS277:HZ1 - Zeatin:O16	2.100000
Zeatin:H29 - A:ASP240:OD1	2.390000
Zeatin:H29 - :ASP240:OD2	2.001000	
Zeatin:C2 - :LYS59:HZ1	2.188000
Zeatin:C15 - :LYS277:H Zeatin:H27 - :LYS277:HZ1	2.105000
1.596000

## References

[1] Mickelbart MV (2015). Nat Rev Genet.

[2] Bailey-Serres J (2012). Plant Physiol.

[3] Bray EA (2000). Responses to abiotic stresses In: Biochemistry and molecular biology of plants.

[4] Bahuguna RN, Jagadish KSV (2015). Environ Exp Bot.

[5] Asthir B, Bhatia S (2014). J Food Sci Technol.

[6] Monjardino P (2005). Crop Science.

[7] He Y, Huang B (2007). Crop Science.

[8] Ferreira S (2006). Annals of Botany.

[9] Demirevska-Kepova K (2005). Biologia Plantarum.

[10] Vierling E (1991). Annual Review of Plant Physiology and Plant Molecular Biology.

[11] Blumenthal C (1990). Australian Journal of PlantPhysiology.

[12] Krishnan M (1989). Plant Physiology.

[13] Song A (2014). International journal of molecular sciences.

[14] Sung DY, Guy CL (2003). Plant Physiology.

[15] Lee JH, Schöffl F (1996). Mol Gen Genet.

[16] Park CJ, Seo YS (2015). Plant Pathol J.

[17] Reddy PS (2010). Mol Gen Genet.

[18] Karlin S, Brocchieri L (1998). J Mol Evol.

[19] Daugaard M (2007). FEBS Lett.

[20] Flaherty KM (1990). Nature.

[21] Flaherty KM (1991). Proc Natl Acad Sci USA.

[22] Zhu XT (1996). Science.

[23] Vogel M (2006). J Biol Chem.

[24] Swain JF (2007). Mol Cell.

[25] Kityk R (2012). Mol Cell.

[26] Zhuravleva A (2012). Cell.

[27] Ahammed G (2014). Curr Protein Pept Sci.

[28] Peleg Z, Blumwald E (2011). Curr Opin Plant Biol.

[29] Xia XJ (2015). J Exp Bot.

[30] Suzuki N (2016). PloS ONE.

[31] Zandalinas SI (2016). J Exp Bot.

[32] Zandalinas SI (2016). BMC Plant Biol.

